# Nanoscale characterization of local structures and defects in photonic crystals using synchrotron-based transmission soft X-ray microscopy

**DOI:** 10.1038/srep24488

**Published:** 2016-04-18

**Authors:** Hyun Woo Nho, Yogesh Kalegowda, Hyun-Joon Shin, Tae Hyun Yoon

**Affiliations:** 1 Department of Chemistry, College of Natural Sciences, Hanyang University, Seoul, 04763, Republic of Korea; 2 Pohang Accelerator Laboratory and Department of Physics, Pohang University of Science and Technology, Pohang, 37673, Republic of Korea

## Abstract

For the structural characterization of the polystyrene (PS)-based photonic crystals (PCs), fast and direct imaging capabilities of full field transmission X-ray microscopy (TXM) were demonstrated at soft X-ray energy. PS-based PCs were prepared on an O_2_-plasma treated Si_3_N_4_ window and their local structures and defects were investigated using this label-free TXM technique with an image acquisition speed of ~10 sec/frame and marginal radiation damage. Micro-domains of face-centered cubic (FCC (111)) and hexagonal close-packed (HCP (0001)) structures were dominantly found in PS-based PCs, while point and line defects, FCC (100), and 12-fold symmetry structures were also identified as minor components. Additionally, *in situ* observation capability for hydrated samples and 3D tomographic reconstruction of TXM images were also demonstrated. This soft X-ray full field TXM technique with faster image acquisition speed, *in situ* observation, and 3D tomography capability can be complementally used with the other X-ray microscopic techniques (i.e., scanning transmission X-ray microscopy, STXM) as well as conventional characterization methods (e.g., electron microscopic and optical/fluorescence microscopic techniques) for clearer structure identification of self-assembled PCs and better understanding of the relationship between their structures and resultant optical properties.

Photonic crystals (PCs) have been considered as one of the most effective materials to control dielectric periodicity^1^,^2^. One of the key properties of PCs is the presence of a photonic band gap (PBG), photonic analogue of the electronic band gap found in semiconductors. PBG arises from periodic modulation of the dielectric function (refractive index) in at least one crystallographic direction^3^. The presence of PBG in these PC materials makes them attractive for the fabrication of various devices and applications, including photovoltaics^2^, metamaterials^4^, waveguides^5^, bio-sensing^6^, photonics^7^, and optoelectronics^8^. Therefore, there have been many efforts to develop techniques for the fabrication of PCs, such as colloidal self-assembly^9^, colloidal crystal templating^10^, and lithographic methods^11^. Among these techniques, the self-assembly of colloidal microspheres is thought as one of the simple, inexpensive and promising routes for fabricating PCs^12^. However, one of the current issues in PC research is the development of appropriate structural characterization methods for PCs and the understanding of the relationship between those crystal structures and resultant optical properties. For instance, as shown in Table 1, various analytical techniques have been applied to characterize PCs, such as scanning electron microscopy (SEM), transmission electron microscopy (TEM), confocal scanning laser microscopy (CSLM), stimulated emission depletion (STED) microscopy and diffraction pattern analysis using laser or X-rays. Although SEM can acquire direct images with high spatial resolution, the observation is limited to the surface of the PCs^13^. TEM is also one of the good candidate techniques, particularly in terms of spatial resolution. However, it is limited to the characterization of PCs with thickness below ~100 nm. Typical PCs with thickness of a few micrometers cannot be observed by TEM due to the short penetration depth of electron. Optical (CSLM, STED) microscopic techniques were also conducted on the structural identifications of three dimensional (3D) self-assembled colloidal crystals. Wei *et al.*^14^ used CSLM to determine the stacking structure and faults in colloidal crystals. However, the diffraction limited spatial resolution of CSLM is not suitable to analyze PCs with submicron-sized colloidal particles. To overcome the limitations of CSLM, Harke *et al.*^15^ used STED microscopy with 43 nm lateral and 125 nm axial resolutions to study the self-assembled PCs. Nonetheless, in those optical/fluorescence microscopy, it is necessary to label PCs with fluorescent dyes and match refractive index of the solvent and particles^14^. These processes may alter the local structures of PCs to a greater extent by exerting the capillary forces on colloidal particles and rendering them unsuitable for the study of self-assembled PCs. To overcome these limitations of optical microscopy (OM), Thijssen *et al.*^16^ used reciprocal space X-ray diffraction technique to identify crystal structure and defects in long-range order. However, this approach cannot probe the local structural variations which are critical to understand photonic crystal growth process. Therefore, a label-free, direct imaging technique with higher penetration depth and better spatial resolution is needed to probe local structural variations and internal defects of thick colloidal PCs.

As demonstrated in Table 1, X-ray microscopy techniques can complement limitations of aforementioned techniques by providing enough penetration depth and spatial resolution, label-free imaging, and capability to probe local structural variations. Compared with the electron microscopy techniques, higher penetration depth of X-ray can allow probing thicker PCs, while shorter wavelength can provide better spatial resolution than optical/fluorescence microscopic techniques without special sample preparation, such as fluorescent labelling or refractive index matching. Therefore, X-ray microscopy has been considered as a promising approach for the characterization of PCs^17^,^18^, and those capabilities have been tested in recent studies using scanning transmission X-ray microscopy (STXM)^19^,^20^. However, structural studies of PCs using full field X-ray microscopy (TXM) at soft X-ray region have not been reported yet.

In this study, we have investigated direct imaging capabilities of a synchrotron-based TXM at water window (500 eV) via characterization of self-assembled polystyrene (PS)-based PCs (up to four layers, approximately 2 μm). Local defects (line and point defects) as well as various crystal structures of PCs (face-centered cubic (FCC), hexagonal close-packed (HCP), and 12 fold symmetry structures) were identified using this synchrotron-based soft X-ray full-field TXM technique with an acquisition time less than ~10 sec/frame.

## Results and Discussion

### Nanoscale characterization of FCC (111) and HCP (0001) mixed structures in PCs

**Figure 1:**
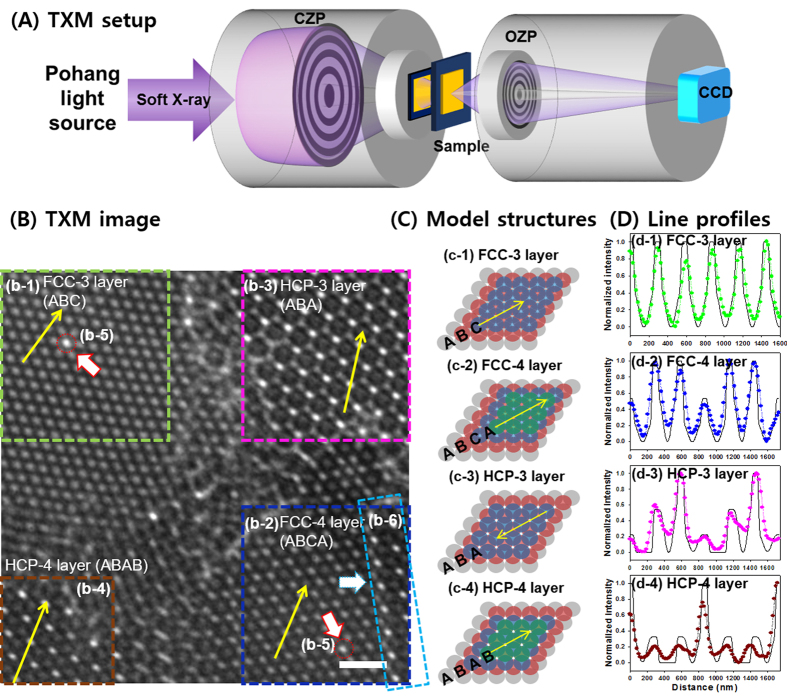
(**A**) Schematic illustration of TXM setup. (**B**) TXM transmission image of self-assembled polystyrene photonic crystals (scale bar of 1 μm). (b-1) Triple- and (b-2) quadruple- layer of FCC indicated by green and dark blue dashed boxes, respectively. (b-3) Triple- and (b-4) quadruple- layer of HCP indicated by pink and brown dashed boxes, respectively. (b-5) Point and (b-6) line defects represented as red solid line arrows with red dashed circles and a light blue arrow with a light blue dashed box, respectively. (**C**) CAD drawn model structures. (c-1) Triple- layered FCC, (c-2) quadruple- layered FCC, (c-3) triple- layered HCP, and (c-4) quadruple- layered HCP crystal structures. (**D**) Comparison of line profiles between FCC (green and blue colored dot lines in d-1 and d-2) and HCP (pink and brown colored dot lines in d-3 and c-4) structures in TXM image and CAD model FCC (black solid lines in d-1 and d-2) and HCP (black solid lines in d-3 and d-4) structures. The position and direction of line profiles of (**D**) are indicated with yellow arrows in (**B**,**C**).

In this study, PS-based PCs were grown on an O_2_ plasma treated, 100 nm thick Si_3_N_4_ window via self-assembly method. Then, various local structures and defects of the PS-based PCs were characterized by the soft X-ray TXM endstation at the 10D beamline of the Pohang Light Source (PLS, Pohang, Republic of Korea). As illustrated in Fig. 1A, the condenser zone plate (CZP) was used to focus incident X-ray on the sample, and the objective zone plate (OZP) was used to magnify the projected image on the charge-coupled device (CCD). When assessing the performance of PCs, the degree of crystal perfection is one of the key parameters. Although the complete details of self-assembly growth mechanisms of PCs are not fully understood yet, it is well recognized that crystal structures and defects are determined by the size ratios, size distributions and volume fractions of each particle, ionic strength of solvent, and the flow rates of solvent compensating for the evaporation from the crystal surface^21^. For instance, the colloidal spheres with minimal ionic interaction are known to arrange in a hexagonally close-packed fashion parallel to the substrate^22^, while further stacking of subsequent hexagonally close-packed layers defines whether the resultant crystal structure is FCC or HCP. Due to the small free energy difference between FCC and HCP crystal structures, it has been reported that the self-assembled growth of PCs often results in a mixture of both structures. However, since it is difficult to distinguish FCC and HCP structures by SEM observing top surface layer without cross-sectional characterizations of the sample, these colloidal crystal structures could be often misinterpreted.

Figure 1B shows typical TXM image of PS-based PCs. The colloidal PS particles were arranged in a close-packed fashion (i.e., FCC in regions b-1 and b-2, and HCP in regions b-3 and b-4) with local defects (i.e., point defect in region b-5 and line defect in region b-6). In regions b-1 and b-2 of Fig. 1B, triple- and quadruple- layered close-packed FCC structures oriented in the (111) plane were found with stacking sequences of ABC and ABCA, respectively. Details of individual particle positions, structures, and differences of number of layers were clearly identified from this TXM image, although the PCs with different stacking sequences (e.g., ABAC or ABCA) were not distinguishable yet from this 2D TXM image only. The FCC and HCP model structures are presented in Fig. 1C, where the circles with grey-, red-, blue-, and green- color indicate the first, second, third, and fourth layer, respectively. In the panel d-1 and d-2 of Fig. 1D, line profiles of the triple- and quadruple- layered FCC structures were displayed (dot lines with green and blue colors, respectively). Then, those measured profiles were compared with simulated profiles (black solid lines). The line profiles measured from TXM images matched well with the simulated profiles of FCC model. For example, the peak-to-peak distance between tetrahedral holes is measured as 291 nm, which is in good agreement with the simulated distance from model structure (i.e., 1/√3 times the diameter of the sphere, 289 nm). TXM images of triple- and quadruple- layered HCP structures were also found in regions b-3 and b-4 of Fig. 1B, respectively. In triple-layered HCP structure (region b-3 of Fig. 1B), the PS spheres in the third layer were placed directly over the PS spheres in the first layer with stacking sequence of ABA. Region b-4 of Fig. 1B also shows a quadruple-layered HCP with stacking sequence of ABAB, where the bright spots (i.e., octahedral hole) were surrounded by 6 completely overlapped PS spheres. The periodicities of the lattice structures were confirmed by the comparisons of the observed and simulated line profiles of triple- and quadruple- layered HCP structures (Panel d-3 and d-4 of Fig. 1D), which matched well for the peak-to-peak distances between octahedral holes in a plane. (i.e., simulated distance is 866 nm, √3 times of the diameter, and measured distance in panel d-3 is 885 nm).

### Nanoscale identification of rarely found local structures in PCs

**Figure 2:**
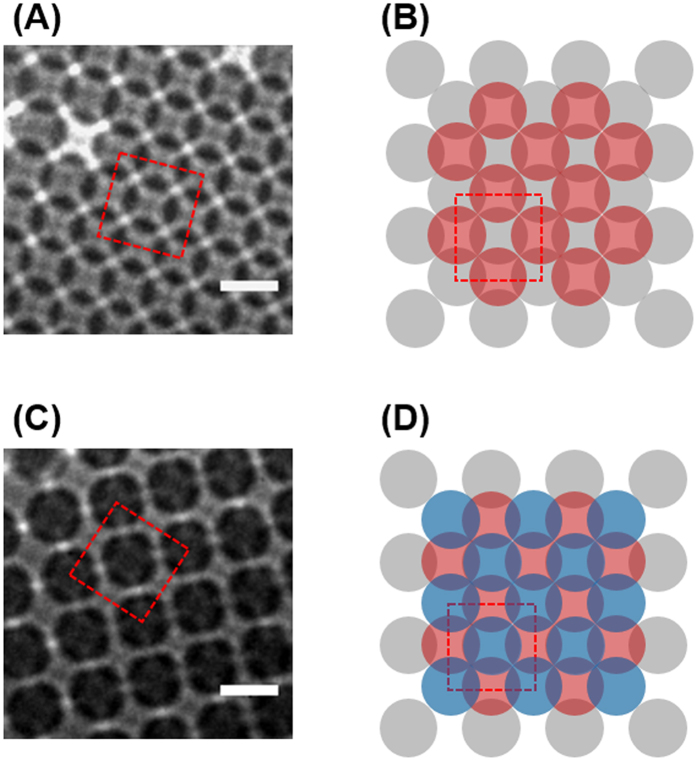
TXM images of (**A**) double and (**C**) triple layered (100) face FCC structures of PCs (scale bar of 1 μm). CAD drawn model structures of (**B**) double and (**D**) triple layers.

**Figure 3:**
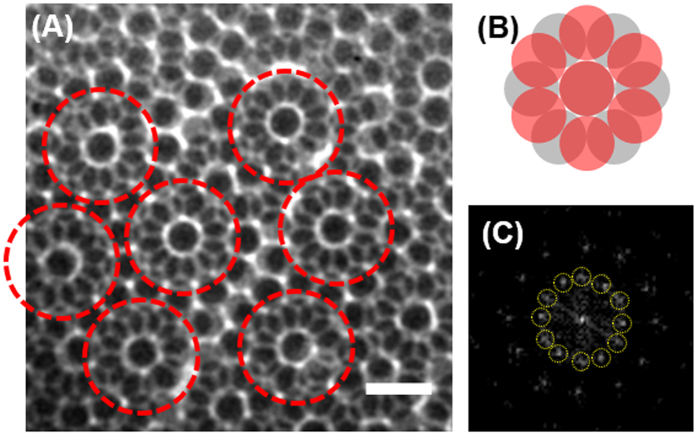
(**A**) TXM transmission image of 12-fold symmetric structure, with 1 μm scale bar. (**B**) Model structure drawn by CAD. (**C**) Fast Fourier transform image of the 12-fold symmetry structure from (**A**).

In addition to close-packed FCC (111) and HCP (0001) structures, (100) face of FCC and 12-fold symmetric structures were also found at the edge of the colloidal PCs or between colloidal crystal domains (Figs 2 and 3). Because of uncontrolled drying process in self-assembly growth, there could be fluctuations in the particle concentration or drying rate, which may induce structural changes in meniscus of colloidal solutions^23^. In Fig. 2, square lattice with an angle of 90° between the two particles are shown as the (100) face of FCC structures. TXM images of the double and triple layered (100) face of FCC are shown in Fig. 2A,C, and the model structures of those are illustrated in Fig. 2B,D. The circles with grey-, red-, and blue- color represents the first, second, and third layer, respectively, and the unit cell is marked as red dash square. In the double layered structure, shown in Fig. 2A, the unit cell length is about 720 nm, therefore the structure resembles FCC (100) face. The theoretical unit cell length (inter-particle distance in same plane) of FCC is about 707 nm, √2 times diameter of the particle. In the triple layered structure shown in Fig. 2C, the darkest spots represent the overlap of the first and third layer.

Figure 3A presents 12-fold symmetric structures formed by superposition of two hexagonally close-packed layers. The dark spots at the center of the red-dashed circles are completely overlapped PS spheres, while the 6 surrounding PS spheres of the first and second layer are rotated 30° to each other. For better understanding of this TXM image, a model of 12-fold symmetric structure is displayed in Fig. 3B, where grey and red circles indicate the first and second layer, respectively. Generally, crystal structures with long-range order (translational periodicity) can be identified by conventional diffraction techniques, while short-range orders can only be identified by the direct imaging techniques capable of probing selected regions, such as TEM-SAED (selected area electron diffraction). However, TEM-SAED technique is not applicable for the PCs with thickness over a few microns. Laser light diffraction have been used to determine the overall structure of PCs. However, it is difficult to probe the local PC structures having short-range orders, such as the 12-fold symmetric structures, due to the relatively large spot size of the laser light source. Therefore, when these conventional diffraction techniques were used, these 12-fold symmetric structures having short-range orders without periodicity is probably misinterpreted as an amorphous structure or other structures. Direct TXM image, as shown in Fig. 3A, is able to identify these unusual local structures with short-range orders. Figure 3C presents fast Fourier transform (FFT) result of the TXM image, in which the surrounding 12 bright spots correspond to the local short-range ordered structure within the red-dashed circles. These results suggest that TXM imaging techniques can be applied to identify rarely-found, local structures of PCs, such as quasi- crystals or intermediate state like a glass transition state of colloidal model systems.

### Nanoscale characterization of point and line defects in PCs

In addition to the types of local structures in PCs, one of the fundamental limiting factors in analyzing PCs is the presence of lattice defects, which are intrinsic to most self-assembly process of PCs. Stacking faults and vacancies are known to affect their optical properties and required to be characterized with minimum modification of the samples. CSLM and STED techniques were often used to study the faulty plane defects in PCs^14^,^15^,^24^,^25^. However, as previously mentioned, these techniques have a potential risk of sample alteration. To overcome these limitations, Hilhorst *et al.*^20^ applied tomographic STXM technique for the study of 3D structure and defects in colloidal silica-based PCs. The tomographic STXM image revealed both local structure (FCC or HCP) and defects by identifying positions of individual particles in PCs^20^. However, synchrotron-based TXM technique has additional advantages over STXM, particularly in terms of acquisition time. For instance, TXM can provide much higher image acquisition speed than STXM technique. Therefore, it is more practical and efficient to use TXM technique when performing identification and characterization of local structures and defects of PCs over wide area or conducting 3D tomographic studies of PCs, since a few tens or hundreds of TXM images should be acquired for these applications within a short observation time. Recently, characterization of 3D colloidal crystals and inverse opal structures has been reported^18^,^26^,^27^ by using hard X-ray TXM of beamline BL01B at National Synchrotron Radiation Research Center (NSRRC) and beamline ID06 at European Synchrotron Radiation Facility (ESRF). However, due to the lower contrast mechanism of hard X-ray TXM for soft materials, it might be difficult to perform highly sophisticated structural analysis of PCs composed of soft materials. In the present study, numerous defects in the PCs were easily and rapidly identified by using soft X-ray TXM techniques. Point defects (region b-5 of Fig. 1B) were observed as bright spots, while region b-6 of Fig. 1B showed a single line defect due to the dislocation and misalignment in the bottom layer.

### Three dimensional tomographic reconstruction of TXM images

**Figure 4:**
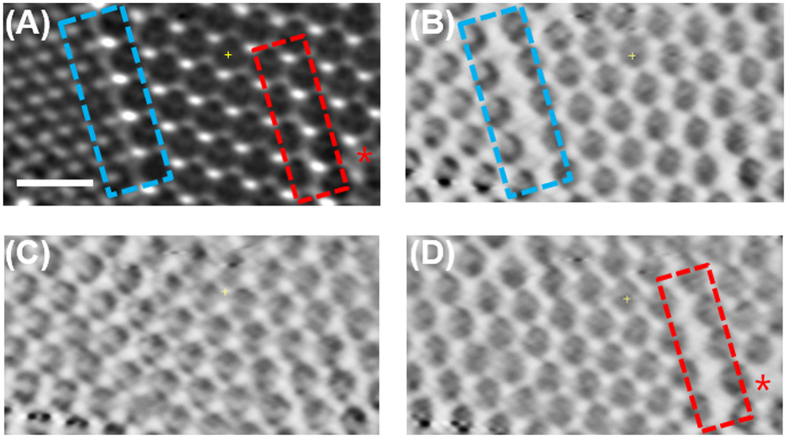
(**A**) TXM transmission image of triple layered PCs (scale bar of 1 μm). Internal line defect shown in blue dash box, and line and point defects on the surface denoted as the red dash box and red star, respectively. (**B**) The bottom (the first layer), (**C**) middle (the second layer) and (**D**) top layers (the third layer) of reconstructed image.

As described above, point or line defects of PCs could be easily found by TXM technique. However, it is also true that we could not determine the 3D location of those line or point defects by using 2D TXM images. Additionally, as mentioned in the previous section, it is also not feasible to determine the stacking sequences of PC structures (e.g., ABAC or ABCA) from only 2D TXM images. Generally, SEM images of cross-sectioned PC samples have been used to identify the 3D locations of point or line defects in PCs. However, this sample preparation process may cause artefacts during destructive cross-sectioning procedure. Therefore, we have applied 3D tomographic reconstruction approach to find 3D locations of line or point defects of PCs and presented in Fig. 4. To reconstruct a complete 3D tomographic image, it is typically necessary to acquire a few tens or hundreds of 2D images with different tilting angles. However, in this study, we have acquired 2D TXM images with 9 different tilting angles and reconstructed 3D tomographic TXM image, which was found sufficient to determine the colloidal layer containing the line or point defects. As shown in Fig. 4B–D, individual layer of PCs could be identified by observing virtually sectioned plane in the reconstructed tomographic image. By using this tomographic technique, we could determine the layers containing various defects without destructive sample preparation process. As shown in Fig. 4A,B, the domain boundary between FCC and HCP was induced by the line defect in the first layer of the PCs (see blue dashed box in Fig. 4A,B). However, this dislocation was not propagated to the second (Fig. 4C) or the third layer (Fig. 4D). In general, line defects formed by successive dislocation of particles can modify the crystal structures of PCs and generate misaligned structures or grain boundaries^28^, which was in good agreement with our observation shown in Fig. 4. These observations demonstrated that the 3D tomographic TXM technique is useful for the characterization of 3D locations of defects in PCs. Additionally, we have confirmed that it is more practical and efficient to use TXM technique, rather than STXM techniques, when characterizing local structures and defects of PCs via 3D tomographic study of PCs, due to the faster image acquisition capability of TXM technique over STXM technique.

### *In situ* observation capability and radiation damages of soft X-ray TXM technique

**Figure 5:**
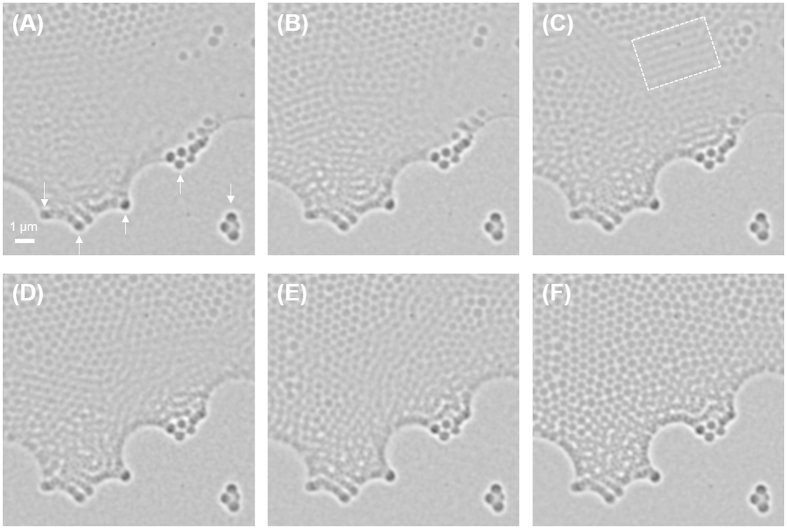
Time-lapse TXM images of 500 nm SiO_2_ particles in hydrated condition. (**A–F**) Drying process of colloids was observed with 13 second interval time. Fixed particles were marked as arrows in (**A**). (**B**) Assembled particles along the meniscus. (**C**) The successive linear migration of suspended particles to fill voids (white dash box). (**D,E**) Reduced blurred regions corresponding to moving colloids in the hydrated condition. (**F**) Completely dried colloids.

Another advantage of soft X-ray TXM imaging at water window is that it allows us to perform *in situ* observation of PCs during their growing process, which may help us to elucidate the mechanism of their growth and defect formation. In order to demonstrate the *in situ* observation capability of TXM for the PCs under hydrated condition, drying process of colloidal particles was monitored in time lapse mode with an interval time of approximately 13 seconds (see Fig. 5). At the edges of the hydrated sample, rearrangement of colloidal particles was consistently observed until the end of drying process. As shown in Fig. 5, thickness of the colloidal solution was not uniform. In thicker regions of the sample, we could not observe the clear shape of suspended particles, probably due to the fast movement of particles by Brownian motion. However, as the drying process continues, evaporation of solvent and resultant capillary action near the meniscus provided the driving force for the successive migration of suspended colloidal particles from thicker regions of the solution to the shallow boundary regions.

When measuring TXM images for PCs and hydrated colloidal particles, no significant radiation damages, such as changes in optical density or morphology induced by mass loss, were observed. There were several reports on the radiation damage in soft X-ray region for the poly(methyl methacrylate) (PMMA), involving mass loss caused by the degradation of carbonyl functional group^29^,^30^. According to those studies, critical radiation dose of PMMA mass loss is 350 eV/nm^3^, while mass loss of PS or SiO_2_-commonly used materials for PCs – by radiation is negligible^29^,^30^. Therefore, we assumed that radiation damage in this TXM study is marginal, mainly due to the fast image acquisition time with low radiation dose.

### Advantages of soft X-ray TXM technique for the characterization of PCs

Similar to the recent STXM studies^19^,^20^, TXM imaging technique at water window can also overcome current limitations of conventional PC characterization methods. Compared with the electron microscopy techniques, higher penetration depth of X-ray can allow probing thicker PCs (about 2 μm, in this study). Meanwhile, shorter wavelength than visible light can provide better spatial resolution than optical/fluorescence microscopic techniques without special sample preparation procedures, such as fluorescent labelling or refractive index matching. In addition, although the TXM setup at the 10D beamline of PLS was optimized for the water window, it is also possible to achieve even higher penetration depth via adapting higher energy regions of soft X-ray (e.g., Si K-edge). Moreover, as described in Table 1, soft X-ray TXM technique provides a few additional benefits over STXM approach. For instance, due to the use of full-field imaging mode rather than scanning mode of STXM, even bending magnet based soft X-ray TXM endstation provided much faster image acquisition speed (about 10 sec/frame) with a relatively larger field of view (FOV, 10 μm × 10 μm with 500 × 500 pixels/image) than that of undulator-based STXM. Since the spatial resolution of TXM is determined by the outer zone width (Δr) of the objective zone plate (~1.22·Δr), faster image acquisition can be achieved without sacrificing the spatial resolution. Moreover, the spatial resolution of STXM depends not only on Δr but also on scanning step size. Therefore, image acquisition with fine steps is needed for higher resolution of STXM images. In a recent study by Schooneveld *et al.*^19^, it took 360 seconds to acquire a STXM image with FOV of 5 μm × 5 μm and spatial resolution of about 30 nm (500 × 500 pixels/image, dwell time of 1 ms/pixel). Further improvement in acquisition time can be achieved for the TXM operating at an undulator source with brighter photon source^31^. Due to this faster acquisition capability of TXM, local structures and defects of PCs can be observed more efficiently in 3D tomography mode or for the wider sample areas, where collections of a few tens or hundreds of TXM images are required.

In this study, we have demonstrated the capability of soft X-ray TXM technique for the nanoscale characterization of various internal structures and defects of PS-based PCs. Point and line defects, FCC, HCP, and 12-fold symmetry structures of PS-based PCs were directly imaged with an acquisition time less than 10 sec/frame, which is faster than those reported for STXM technique^19^. In addition to these benefits of TXM techniques for the characterization of PCs, *in situ* observation capability for hydrated samples and 3D tomographic reconstruction of TXM images were demonstrated. Thus, we believe that these advantages of TXM technique can be complementally used with other X-ray microscopic techniques (i.e., STXM) and conventional analytical tools for better characterization of self-assembled colloidal PCs.

## Experimental Methods

### TXM experimental setup

Synchrotron based X-ray transmission images were acquired at 500 eV (wavelength of 2.48 nm) with over 10^10^ photons/s (0.1% BW) photon flux from the 10D beamline of the Pohang Light Source (PLS, Pohang, Republic of Korea)^32^. Detailed description and schematic illustration of the TXM setup used in this study were presented in recent publications^32^,^33^. The incoming X-ray was focused on the sample by a condenser zone plate (CZP, 2 mm diameter, 110 nm outermost zone width), and the transmitted X-ray was magnified with an objective zone plate (OZP, 84 μm diameter, 38 nm fine zone width). A charge-coupled device (CCD, Peltier-cooled, in-vacuum back-illuminated 16 bit CCD, 1024 × 1024 pixels, 13 μm pixel size, Princeton Instruments, PIXIS-XO 1024B) was used to capture the projected image, and was installed 1.5–1.8 m from the OZP. Si_3_N_4_ membrane windows were used to maintain the vacuum condition for optical elements except OZP. OZP and sample mounting stages were open to the atmosphere. The free space between the CZP side window and OZP was 3–4 mm, and the PCs on Si_3_N_4_ membrane was mounted in this free space. Helium gas was infused from beneath the sample to minimize X-ray attenuation by N_2_, O_2_, CO_2_, etc. in air.

### Fabrication of photonic crystals for TXM observation

Colloidal PCs were self-assembled by drop casting on a Si_3_N_4_ membrane substrate. Commercially available polystyrene (PS) with diameter of 500 nm (25 mg mL^−1^ with a polydispersity of 3%; Polysciences, Warington, PA, USA) was used to make the opal-like structures on a 100-nm-thick Si_3_N_4_ membrane substrate (NX5150C, Norcada Co., Edmonton, AB, Canada). The substrate was treated with O_2_ plasma (100W, 0.2–1 mbar, 45 s; CUTE, Femto Science, Gyeonggi-Do, Republic of Korea) for cleaning and hydrophilic surface modification. Then, 5 μL of the PS colloidal solution was dropped on the Si_3_N_4_ substrate and slowly evaporated at room temperature to obtain colloidal crystalline structures in large area.

### Image processing and tomographic reconstruction

Measured TXM images were cropped and further processed by Java-based image processing and analysis software (ImageJ 1.41n, http://rsb.info.nih.gov/ij, Wayne Rasband, NIH, USA). The IMOD tomography package was used to align and reconstruct of images acquired from −30° to 50° with step 10°. Defect points in PCs were used for aligning and tracking to 3D reconstruct. Reconstruction was performed by a weighted back-projection algorithm in IMOD package.

## Additional Information

**How to cite this article**: Nho, H. W. *et al.* Nanoscale characterization of local structures and defects in photonic crystals using synchrotron-based transmission soft X-ray microscopy. *Sci. Rep.* **6**, 24488; doi: 10.1038/srep24488 (2016).

## Figures and Tables

**Table 1:** Comparison of various microscopic techniques used for characterization of photonic crystals.

Important characteristics	Optical microscopy (OM)		Electron microscopy (EM)	Soft X-ray microscopy (XM)	
	Confocal scanning laser microscopy (CSLM)^19^,^24^	Stimulated emission depletion microscopy (STED)^15^,^25^	Field emission scanning electron microscopy (FE-SEM)^19^	Scanning transmission X-ray microscopy (STXM)^19^	Full field transmission X-ray microscopy (TXM)^c^
Field of view (μm)	Up to 50 × 50	Up to 38 × 38	6000 at 25X	5 × 5 (adjustable)	10 × 10
Sample thickness (μm)	10–100	10–100	0.010–0.1	~0.6^a^, ~6.5^b^	~2
Acquisition speed (sec/frame)	0.25–10	0.0125–0.1	A few tens of second	360 (500 × 500 pix/image)	~10 (500 × 500 pix/image)
Spatial resolution (nm)	200–1000	50–1000	2–10	30	46
Sample preparation	Fluorescent labelling	Fluorescent labelling	Au or Pt coating	Not required	Not required
*In situ* capability	Very good	Very good	Poor	Good	Very good

^a^Advanced Light Source (ALS), Undulator beamline (11.0.2), measured at 315 eV^19^.

^b^Canadian Light Source (CLS), Undulator beamline (SM), measured at 1,845 eV^19^.

^c^Pohang Light Source (PLS), Bending magnet beamline (10D), measured at 500 eV [in this study].
